# Direct patient costs for drugs and consumables at fifteen health facilities in Southern Madagascar, a secondary analysis of patient invoices

**DOI:** 10.1371/journal.pone.0311253

**Published:** 2024-10-10

**Authors:** Mara Anna Franke, Anne Neumann, Kim Nordmann, Daniela Suleymanova, Onja Gabrielle Ravololohanitra, Samuel Knauss, Julius Valentin Emmrich

**Affiliations:** 1 Global Digital Health Lab at Charité Center for Global Health, Charité –Universitätsmedizin Berlin, Berlin, Germany; 2 Ärzte für Madagaskar e.V., Leipzig, Germany; 3 Rheinisch-Westfälische Technische Hochschule Aachen, Aachen, Germany; 4 Paris Institute of Political Studies, Paris, France; 5 Doctors for Madagascar, Antananarivo, Madagascar; 6 Heidelberg Institute of Global Health, Heidelberg University, Heidelberg, Germany; 7 Berlin Institute of Health at Charité - Universitätsmedizin Berlin, Berlin, Germany; University of KwaZulu-Natal, SOUTH AFRICA

## Abstract

**Background:**

Financial risk protection in health is a key objective of the Sustainable Development Goals. However, financial risk protection mechanisms are limited, especially in low-income countries, such as Madagascar. To design effective financial risk protection mechanisms, solid and reliable data on the costs patients incur when seeking care are essential. With this study, we therefore aim to describe medical costs for drugs and consumables for patients as well as model the likelihood of catastrophic health expenditure at fifteen health facilities in Southern Madagascar.

**Methods:**

We conducted a costing analysis of patient invoices from fifteen health facilities (four primary and eleven secondary facilities) in Southern Madagascar, including public, private, and faith-based facilities. We included invoices from patients accessing care for life-threatening conditions, accidents and injuries, paediatric, or maternity care between February 2021 and July 2022. Costing data were limited to costs for drugs and consumables. We used regional household expenditure data from a representative household survey to calculate the incidence of catastrophic health expenditure in our sample.

**Results:**

We analysed data from 9,855 cases, including 4,980 outpatient cases, 3,447 inpatient cases without surgical intervention, and 1,419 surgical cases. The average patient cost for drugs and medical consumables across all cases was USD 39.52 (range: USD 0.13–1,381.18, IQR: USD 9.07–46.91). Average costs for surgical treatment were USD 119.33 (range: USD 8.10–522.88, IQR: USD 73.81–160.49), for inpatient treatment USD 47.07 (range: USD 1.82–1,381.19, IQR: USD 22.38–58.91), and for outpatient treatment USD 11.73 (range: USD 0.15–207.79, IQR USD: 6.00–15.53). On average patients at faith-based facilities paid USD 47.20 (range: USD 0.49–530.33, IQR: 10.74–58.54), USD19.47 (range: USD 0.40–1,381.23, IQR: 6.77–24.07) at private facilities, and USD 34.65 (range: USD 0.58–245.24, IQR: USD 6.08–60.11) at public facilities. Patients requiring surgical care were most likely to experience catastrophic health expenditure and average costs for maternity care were significantly higher than for other patient groups.

**Conclusions:**

Financial risk protection schemes in Madagascar, such as the national UHC policy, and the national solidarity fund, as well as interventions by non-governmental and multilateral organisations, need to focus on surgical cases and maternity care to protect vulnerable populations from catastrophic health expenditures for life-threatening conditions, accidents and injuries, and maternity and paediatric care.

## Introduction

Universal health coverage, which includes the protection from financial hardship when accessing medical care is a key goal of the sustainable development goals (SDGs) [1]. However, many health systems around the globe, particularly those in low-and middle-income countries struggle to achieve this goal. Especially in Sub-Saharan Africa (SSA), a large part of healthcare delivery is financed through out-of-pocket (OOP) payments [2] OOP payments (i.e. payments that are made with an individual’s own cash, whether or not they are reimbursed) have an elevated risk of causing catastrophic health expenditure (CHE; i.e., expenditure on medical services that exceed a household’s capacity to pay) [3,4]. Globally, CHE is common. It is estimated that in 2019 alone more than a billion people incurred catastrophic or impoverishing health expenditures worldwide [5]. Extreme poverty poses a risk to an individual’s health, as poverty is a key social determinant of health [6]. Individuals living in extreme poverty are less resilient to future financial shocks, including health-related ones [6]. Thus, CHE can cause an ongoing downward spiral, perpetuating worse health outcomes and extreme poverty, which can hamper overall development prospects [5].

In Madagascar, OOP payments account for approximately 40% of the country’s healthcare expenditure [7]. The island nation of approximately twenty-nine million people is one of the least developed countries globally [8,9]. More than 80% of the population lives in extreme poverty (below USD 2.15 /day, 2017 purchasing power parity) [8] The Malagasy government laid out a plan for universal health coverage (UHC) in its national UHC strategy in 2015 [10]. The strategy foresees free medical care for the population across the health sector, with a particular focus on the poorest strata of society and on cases requiring urgent life-saving and surgical care [10].

In reality, however, financial risk protection schemes for healthcare are almost non-existent in Madagascar; only 2% of the population is covered through some form of health insurance scheme and mechanisms such as the national equity fund that is supposed to offer financial support for destitute patients are largely non-functional [7,11]. In the most recent census, financial reasons were the most commonly cited barrier in accessing healthcare, especially among rural populations [12].

To design effective financial risk protection mechanisms, there is a need for accurate costing data. Equally non-governmental organisations (NGOs), multilateral organisations and private entities (such as insurance companies) involved in financial risk protection in the absence of comprehensive national policies require accurate costing data for programme development, resource allocation and priority setting. In Madagascar, such data is extremely limited. To our knowledge, only three studies have examined the direct costs of medical care for patients [13–15]. All three of these studies focus exclusively on maternal and neonatal or paediatric care and do not account for costs of care for other conditions [13–15]. One study draws on data from the central highland region of Madagascar, and the other on data from a university hospital in the north of the country [14,15]. Both of these studies draw exclusively on data from public facilities [14,15]. Only one study uses data from the rural south of the island, which is most heavily affected by extreme poverty [12,13]. This is also the only study that reports data from faith-based healthcare providers [13]. Costing data for patients requiring medical care beyond maternal and paediatric care, and for patients seeking care in the South of the Island, is sorely lacking.

In this study, we aim to describe the costs incurred by individuals seeking urgent medical care for potentially life-threatening diseases, accidents and injuries, maternity, and paediatric care at public, private, and faith-based primary and secondary facilities in Southern Madagascar. We draw on data from an NGO-initiated cash transfer intervention conducted from February 2021 to July 2022 at fifteen healthcare facilities across seven regions of Southern Madagascar, including public, private, and faith-based primary and secondary facilities.

## Methods

### Study setting

This study draws on observational data from seven regions in Southern Madagascar: Androy, Anosy, Atsimo-Andrefana, Atsimo-Atsinanana, Menabe, Haute Matsiatra, and Vakinankaratra.

Poverty rates in these regions are high, more than 78% of the population live in extreme poverty in Vakinankaratra, rising to over 95% in Androy [12]. The majority live in rural areas and live off subsistence farming [12]. The health system in Madagascar is organised in four tiers, with community health workers providing care at the community level. Basic health centres (Centres de santé de base, CSBs) provide primary care, such as uncomplicated deliveries, vaccination services, etc. Secondary care is only available at district and regional referral hospitals or private/faith-based secondary facilities. Tertiary care is offered by only a few hospitals, which are often located in urban centres and regional capitals. There is no formal referral system between facilities, and patients are generally responsible for organising their own transport to and between health facilities. The Malagasy healthcare system largely functions on OOP payments, some care, e.g. tuberculostatic medication is offered free of charge due to external funding, e.g. from the Global Fund. This is however the exception, and most health facilities require patients to pay for care upfront before initiating treatment.

Access to healthcare is severely limited, including, but not exclusively due to financial reasons; catastrophic road conditions, geographical barriers, and limited healthcare-related knowledge of the population negatively affect access to vital healthcare services [11,12]. The south of Madagascar frequently experiences by natural disasters and extreme weather events, impacting food security in the region. In 2020 and 2021 a severe famine affected the south of Madagascar, putting over two million people into acute food insecurity [16]. At the same time, the COVID-19 pandemic hit Madagascar, with the first case being registered in mid-March 2020 [17]. Subsequent lockdowns and the strained economic situation caused by the pandemic further increased poverty in the country [18].

In this context, the NGO Doctors for Madagascar [19] implemented a conditional digital cash transfer intervention aimed at reducing financial barriers to care at fifteen health facilities, including private, public, and faith-based facilities. Table 1 below shows the participating facilities.

**Table 1:** Health facilities in Southern Madagascar where the conditional digital cash transfer intervention to encourage healthcare seeking was implemented in 2021–2022, as well as facility type, and services offered.

Region	Municipality	Facility	Ownership	Level of care offered
Androy	Tsihombe	CHRD Tsihombe	public	Secondary care
Androy	Ambovombe	CHRR Ambovombe	public	Secondary care
Anosy	Tolagnaro	Centre médical	private	Primary care
Anosy	Tolagnaro	Dispensaire Sahan’i Maria	private, faith-based	Primary care
Anosy	Tolagnaro	SALFA Bazaribe	private, faith-based	Primary care
Anosy	Tolagnaro	Clinique Avotr’Aina	Private, faith-based	Primary care
Anosy	Manambaro	SALFA Manambaro	private, faith-based	Secondary care
Anosy	Tolagnaro	SILOAMA	private	Secondary care
Atsimo-Andrefana	Toliara	SALFA Toliara	private, faith-based	Secondary care
Atsimo-Andrefana	Ejeda	SALFA Dr. Quanbeck Ejeda	private, faith-based	Secondary care
Atsimo-Andrefana	Ampanihy	CHRD Ampanihy	public	Secondary care
Atsimo-Atsinanana	Farafangana	Clinique catholique Ambatoabo	private, faith-based	Secondary care
Menabe	Morondava	SALFA Morondava	private, faith-based	Secondary care
Haute Matsiatra	Fianarantsoa	SALFA Fianarantsoa	private, faith-based	Secondary care
Vakinankaratra	Antsirabe	SALFA Andranomadio	private, faith-based	Secondary care

### Intervention

The intervention, named Tosik’aina (“vital subsidy”), covered 80% of patients’ costs for drugs and medical consumables (i.e. disposable, single-use medical products such as syringes or bandages) for eligible patients seeking care at a participating facility during the intervention period. The costs covered through the intervention were limited to medical consumables and medication costs only. Costs for medical consultations, laboratory fees (apart from laboratory consumables), hospitalisation, as well as indirect costs such as transportation costs were not covered by the intervention due to requirements set by the agency funding the project. These costs were covered by the patients themselves. The project lasted from February 2021 to July 2022. Facilities could register their interest to participate in the project with the implementing NGO which then chose facilities based on previous collaboration experience and geographical location, favouring facilities that served underserved and/or rural populations with high poverty rates. Participating facilities were onboarded on a rolling basis, meaning that not all facilities participated in the intervention for the whole duration. Patients were eligible for the partial cost coverage scheme if they fell into one of four categories: i) patients seeking care for acutely life-threatening conditions, ii) patients seeking care for accidents or injuries, iii) patients seeking care during pregnancy, childbirth or postpartum, and iv) children under five years of age. The decision of whether an eligible patient was included in the program was made by the treating physician. Patients could refuse to participate at any point.

If a patient was eligible to receive partial cost coverage and consented to participate in the program, a claim was filed by the health centre at which the patient was treated. This claim was vetted for correctness by a trained medical team before being cleared for reimbursement. All claims were reimbursed using mobile money, through the digital mTOMADY platform, the largest digital healthcare payment platform in Madagascar.

### Data source

For this study, we used data filed through the mTOMADY claim system. The individual claims contained information about patient symptoms, their sociodemographic characteristics (e.g., age, gender, occupation, household size), their diagnoses, and the costs they incurred for medication and consumables. Costs for other direct and indirect medical expenses were not included in the data.

### Data collection and data entry

Data were first accessed for research purposes on October 14^th^, 2022. Relevant data, such as patient age, patient sex, patient category (acutely life-threatening condition, accidents and injuries, maternity care, paediatric care) treating facility, treatment type (outpatient, inpatient, surgical, non-surgical), patient diagnosis and treatment costs), were transcribed into a standardised Excel data sheet from the photos of prescriptions and invoices, and patient charts and medical data saved on the mTOMADY platform by a research assistant. These invoices stated the full amount of direct costs for medication and consumables incurred by patients, even though only 80% of the costs were covered by the implementing NGO. The full costs for medication and consumables were used for analysis in this study. All data were anonymised in the process. The data were regularly checked for quality, inconsistencies, and plausibility by two independent researchers who sought clarification from the implementing team whenever there were inconsistencies in the data.

All diagnoses were grouped according to the ICD-11 Classification of Diseases [20] by a qualified medical doctor and all classifications double checked by two further licensed doctors with long-term experience in Madagascar. Claims that were declined by the medical team assessing claims for correctness and completeness during the intervention, were excluded from this analysis. Claims for patients presenting with more than one diagnosis and claims that could not be classified according to the ICD-11 classification were equally excluded from the sample. Out of 12,305 claims, we excluded a total of 978 claims (309 because claims were declined, and 669 because the given diagnosis could not be classified into an ICD-11 category). In case of multiple diagnoses for the same patient, the approach depended on the type of diagnoses. In cases where one or more diagnoses could be attributed to one primary diagnosis (e.g. anaemia following postpartum haemorrhage), the patient was assigned the ICD-11 code of the primary diagnosis (e.g. postpartum haemorrhage). Where such a classification was not possible as there was no direct causal relationship between diagnoses (e.g. malaria and prostate hyperplasia), patients were classified under “multiple” and stratified by the number of diagnoses they presented with.

### Data analysis

We defined catastrophic health expenditure based on annual household expenditure collected from the representative household census survey conducted in 2010, which is the last available data set for the study region detailing household expenditure [21]. We adjusted the 2010 expenditure data for inflation to reflect 2022 purchasing power [22]. We set two thresholds for catastrophic health expenditure: 10% of annual household expenditure and 25% of annual household expenditure [23]. The regional cut-off values are shown in Table 2 below.

**Table 2:** Individual annual consumption, average household size, household annual consumption, and catastrophic health expenditure thresholds at 10% and 25% per region, taken from a nationally representative survey (Enquête auprès des menages, 2010)^21^ and adapted for inflation to reflect purchasing power in 2022^21^.

Region	Individual annual consumption (REGIONAL AVERAGE, IN ARIARY)	Average household size^21^	Household annual consumption (in Ariary) ^21^	CHE-threshold at 10% (in Ariary)	CHE- threshold at 25% (in Ariary)
**Androy**	89,146	5.6	499,219	49,922	124,805
**Anosy**	144,795	4.9	709,495	70,950	177,374
**Atsimo-Andrefana**	151,323	4.8	726,352	72,635	181,588
**Atsimo-Atsinanana**	110,217	5.8	639,259	63,926	159,815
**Haute Matsitra**	147,316	5.5	810,239	81,024	202,560
**Menabe**	228,988	4.9	1,122,048	112,205	280,512
**Vakinankaratra**	199,543	5.1	1,017,670	101,767	254,418

For the conversion from Ariary to United States Dollars (USD), we used the annual average exchange rate for 2022, where one USD equals 4,096.12 Ariary [23].

We used ANOVA and Tukey’s HSD to assess differences across the mean patient expenditure across the different patient categories (accidents and injuries, maternal and paediatric care, and potentially life-threatening disease) and types of facilities where patients received treatment. We conducted logistical regression analyses to analyse patient and treatment characteristics that influenced the likelihood of catastrophic health expenditure. All analyses were conducted in R Studio Version 2023.06.1 [24] and a p-value of below 0.05 was considered statistically significant.

### Ethical approval

Ethical approval for this study was obtained from the University of Heidelberg Ethics Committee under registration number S-982/2021 on the 16^th^ of August 2022. As data were anonymised before being passed to the research team and were secondary, the ethics committee waived informed consent for the data source. In addition, we obtained formal approval to conduct this secondary analysis of de-identified NGO data from the district health offices (a regional sub-division of the Malagasy Ministry of Health) in all regions from which data were included in the study.

## Results

### Sample description

The final study sample analysed included 11,327cases, consisting of 6,887 (60.8%) women and 4,429 (39.1%) men. For twelve patients no gender was given. The average age was 29.7 years (range: 0–95 years, IQR: 15–42 years).1,352 (11.9%) cases were children under 5, 1,380 (12.1%) cases related to pregnancy and childbirth, 306 (2.7%) were cases of accidents and injuries, and 8,289 (73.2%) cases of potentially life-threatening diseases (such as malaria, appendicitis, or tuberculosis). 6,458 (57.0%) patients were treated at faith-based facilities, 3,666 (32.4%) at private, and 1,203 (10.6%) at public facilities.

Most patients received outpatient care (5,671, 50%), 4,154 (36.7%) received inpatient care without a surgical intervention and 1,495 (13.1%) received inpatient care as well as a surgical intervention.

The majority of patients (9,111/11,327, 80.4%) presented with one diagnosis. 1,848 (16.3%), 353 (3.1%), and 15 (0.1%) patients presented with two, three, or four diagnoses respectively.

The most common diagnosis in the sample was malaria with a total of 2,329 cases, followed by typhoid fever (588 cases) and delivery (515 cases).

### Patient costs

The average patient cost for drugs and medical consumables across the whole sample was 161,911 Ariary (USD 39.52; IQR: 37,200–192,500 Ariary (USD 9.07–46.91). These ranged from 2,000 Ariary (USD 0.13) to 5,657,680 Ariary (USD 1,381.18).The average costs for medication and consumables for outpatient treatment were USD 11.73 (range: USD 0.15–207.79, IQR: USD 6.00–15.53). Average costs for medication and consumables for inpatient non-surgical treatment were USD 47.07 (range: USD 1.82–1,381.19, IQR: USD 22.38–58.91), and for inpatient surgical treatment USD119.33 (range: USD 8.10–522.88, IQR: USD 73.81–160.49).The average treatment costs for patients treated for accidents and injuries were USD 46.53 (range: USD 0.80–341.71, IQR: USD 12.16–60.23). The average treatment costs for maternal care were USD 67.71 (range: USD 1.24–522.90, IQR: USD 17.86–117.17). Average treatment costs for paediatric care were USD 21.31 (range: USD 0.50–329.62, IQR: USD 6.04–21.28). The average costs for patients being treated for potentially life-threatening conditions were USD 37.62 (range: USD 0.50–1,381.2, IQR: USD 9.19–29.48). The average treatment costs for maternity care were significantly higher than for any other patient group (ANOVA results: F (11321) = 208.3, p-value < 0.01). The results of Tukey’s HSD test are reported in Table 3 below.

**Table 3:** Results of Tukey’s HSD test for comparing mean patient costs across categories (maternity care, paediatric care, accidents and injuries, acutely life-threatening conditions). The sample includes patients receiving outpatient, inpatient, surgical and non-surgical care.

COMPARISON	DIFFERENCE (IN USD)	95% CONFIDENCE INTERVAL	P-VALUE
**Maternity Care / Accidents and Injuries**	21.19	13.00–29.38	< 0.001
**Accidents and injuries / Paediatric care**	25.18	16.98–33.38	< 0.001
**Accidents and injuries / Potentially life-threatening diseases**	8.93	1.39–16.47–20.01	0.012
**Maternity care / Paediatric care**	46.37	41.41–51.33	< 0.001
**Maternity care / Potentially life-threatening diseases**	30.12	26.35–33.89	< 0.001
**Potentially life-threatening diseases / Paediatric care**	16.25	12.45–20.05	< 0.001

The average patient costs at faith-based facilities were USD 50.21 (range: USD 0.49–530.33, IQR: USD 12.01–68.65). At private facilities, patients paid an average of USD 22.42 (range: USD 1.27–1,381.23, IQR: USD 7.20–28.43). The mean patient costs at public facilities were USD 34.34 (range: USD 0.58–245.24, IQR: USD 7.06–54.04). The ANOVA revealed these differences to be statistically significant (F (11,322) = 365.4, p-value < 0.01). We used Tukey’s post-hoc test to further explore the differences in costs between facilities. Costs at private facilities were significantly lower than at faith-based facilities (Mean difference = -27.79, 95% CI [-30.23, -24.36], p < .001) and public facilities (Mean difference = -11.92, 95% CI [-15.84, -8.01], p < .001). Additionally, public facilities demonstrated significantly higher patient costs compared to faith-based facilities (Mean difference = 15.87, 95% CI [12.17, 19.57], p < .001).

To account for the different services provided by different types of facilities, we further analysed the difference in patient costs across facility types, disaggregated by the type of treatment provided. Table 4 below illustrates the patient costs across different facilities for different types of treatment provided.

**Table 4:** Mean patient costs, along with ranges and interquartile ranges, disaggregated by treatment type (outpatient, inpatient non-surgical, and surgical care) and facility type (faith-based, private, and public), obtained from 15 health facilities in Southern Madagascar. All costs are expressed in USD.

	Average costs for outpatient care	Average costs for inpatient non-surgical care	Average costs for surgical care
Faith-based Facilities	13.21 (0.59–207.83, IQR: 6.84–18.42)	54.99 (1.81–530.33, IQR: 23.39–67.87)	142.77 (9.35–522.94, IQR: 105.01–175.36)
Private facilities	10.74 (1.27–73.88, IQR: 6.10–12.41)	45.01 (2.91–1,381.23, IQR: 24.85–52.00)	86.16 (21.87–132.28, IQR: 39.70–127.18)
Public facilities	5.78 (0.58–36.57, IQR: 2.87–7.39)	27.87 (2.15–164.99, IQR: 11.77–37.28)	68.81 (8.05–245.24, IQR: 50.75–79.18)

The results of the ANOVA and subsequent Tukey’s test revealed significant main effects of both facility type (F(11,313) = 746.2, p < .001) and treatment (F(11,313) = 5,883.2, p < .001) on treatment costs.

**Fig 1:**
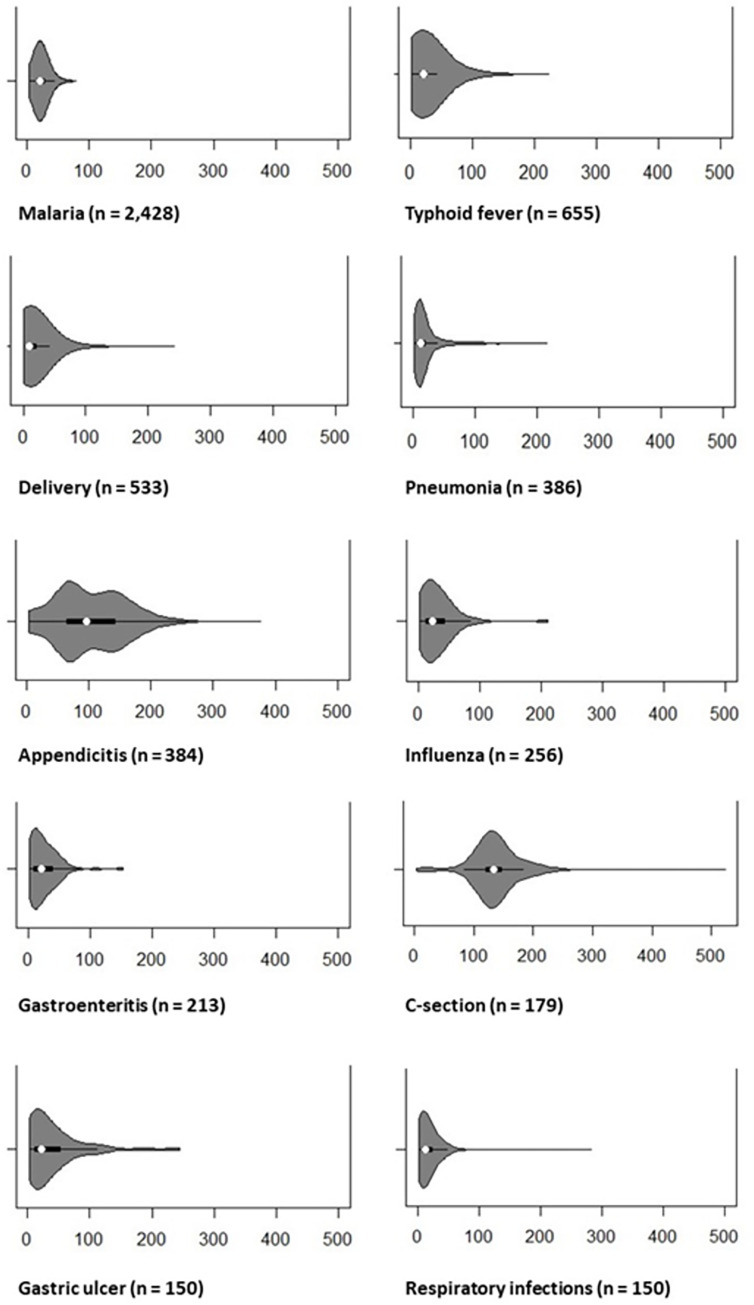
Violin plot of patient costs for the ten most common diagnoses in the study sample expressed in USD. White points represent mean, black boxes interquartile ranges.

Fig 1 below shows the ten most common diagnoses and their average patient costs for drugs and consumables expressed as violin plots. The costs are given in USD.

The average patient costs for patients with one diagnosis were USD 39.32 (range: USD 0.49–1,381.16, IQR: USD 8.67–44.94). For patients with two diagnoses, they were USD 38.08 (range: USD 0.56–340.32, IQR: USD 10–47.1) and for those with three diagnoses average costs amounted to USD 50.57 (range: USD 3.42–347.81, IQR: USD 18.59–71.22). Patients with four diagnoses incurred average costs of USD 119.89 USD (range: USD 58.68–329.63, IQR: USD 70.17–71.24).

### Catastrophic health expenditure

Overall, 6,012 (53.1%) and 3,013 (26.6%) patients incurred catastrophic health expenditures at the 10% and 25% threshold, respectively. A total of 3,847, women incurred CHE at 10%-threshold and 1,915 at 25%-threshold, representing 56% and 27% of all women in the sample, respectively. 2,159 and 1,094 men incurred CHE at 10%- and 25%-threshold respectively, constituting 49% and 51% of all men.

Patients who received inpatient, surgical treatment were most likely to incur CHE.

While only 1,076 (18.9%) of outpatients incurred CHE at the 10%-threshold and only 0.1% (n = 66) at the 25%-threshold, 1,473 (98.5%) and 1,394 (93.3%) patients that received surgical treatment incurred CHE at 10- and 25%-threshold respectively.

Among patients that received inpatient non-surgical treatment, 3,459 (80.3%) and 1,550 (37.3%) incurred CHE at the 10%- and 25%-threshold respectively.

A point of note is that 60%% and33% of people with TB incurred CHE at the 10% and 25% threshold.

Table 5 below shows the likelihood of incurring catastrophic health expenditure for all diagnoses with over 50 cases in our sample.

**Table 5:** Diagnoses with over 50 cases in our sample (total sample n = 9,855), ranked by frequency and rate of catastrophic health expenditure per diagnosis. The sample includes patients receiving outpatient, inpatient, surgical and non-surgical care.

ICD-11 Code (n, total)	Mean costs in USD (IQR)	CHE at 10% threshold (n)	CHE at 25% threshold (n)
**Malaria without parasitological confirmation (2,329)**	17.02 (5.88–20.41)	27.5% (641)	8.5% (198)
**Typhoid fever, unspecified (588)**	27.48 (16.37–26.71)	67.2.3% (391)	13.8% (15)
**Delivery, unspecified (515)**	22.33 (15.60–27.08)	60.9% (314)	3.2% (17)
**Pneumonia, organism unspecified (362)**	19.64 (7.23–19.71)	31.2% (113)	9.4% (34)
**Appendicitis, unspecified (369)**	101.50 (64.15–141.66)	92.4% (345)	90.2% (333)
**Influenza (205)**	13.29 (6.70–16.91)	24.8% (51	1.9.6% (4)
**Gastroenteritis or colitis without specification of infectious agent (187)**	27.44 (10.18–39.07)	55.6% (104)	18.2% (34)
**Single delivery by caesarean section (169)**	139.15 (121.51–146.30)	98.8% (167)	97.7% (165)
**Respiratory infections, not elsewhere classified (137)**	19.62 (6.79–25.01)	30.7% (44)	6.7% (9)
**Ulcer of stomach or duodenum (138)**	39.92 (12.46–52.33)	57.9% (80)	26.8% (37)
**Essential hypertension, unspecified (120)**	35.89 (11.54–51.69)	64.2% (77)	26.7% (32)
**Injury, poisoning, or certain other consequences of external causes (129)**	33.76 (11.48–42.62)	53.5% (69)	24% (31)
**Hernias, unspecified (130)**	95.43 (62.55–122.45)	97.7% (127)	92.3% (120)
**Tuberculosis, unspecified (91)**	49.14 (15.67–77.62)	65.9% (60)	36.3% (33)
**Bronchitis, unspecified (90)**	20.77 (8.73–22.91)	26.1% (27)	6.3% (7)
**Ectopic pregnancy, unspecified (90)**	138.07 (94.46–174.28)	100% (90)	97.8% (89)
**Acute pharyngitis, unspecified (84)**	16.12 (8.44–19.57)	30.9% (26)	3.6% (3)
**Injuries to the head (81)**	52.06 (19.80–73.74)	77.8% (63)	53.1% (43)
**Schistosomiasis (77)**	30.87 (9.46–36.32)	49.3% (38)	19.5% (15)
**Urinary tract infection, site not specified (75)**	16.04 (8.53–30.88)	40% (30)	10.7% (8)
**Diarrhoea (72)**	22.11 (7.76–33.08)	43.1% (31)	13.8% (10)
**Salpingitis and oophoritis, unspecified (71)**	22.27 (7.34–22.75)	28.2% (20)	9.9.%(7)
**Stroke not known if ischaemic or haemorrhagic (73)**	75.90 (24.72–105.78)	84.9% (62)	56.2% (41)
**Pain localised to upper abdomen (63)**	22.06 (7.53–34.11)	49.2% (31)	22.2% (14)
**Abdominal or pelvic pain (63)**	24.24 (7.58–23.09)	36.5% (23)	15.9% (10)
**Cough (61)**	10.79 (2.60–7.54)	11.5% (7)	6.6% (4)
**Leiomyoma of uterus (61)**	149.72 (104.86–191.32)	96.7% (59)	91.8% (56)
**Other or unspecified ovarian cysts (60)**	93.54 (36.78–146.59)	80% (48)	75% (45)
**Obstructed labour due to foetopelvic disproportion, unspecified (59)**	156.80 (107.82–105.18)	100% (59)	100% (59)
**Infection, unspecified (59)**	29.91 (12.24–40.26)	55.9% (33)	23.7% (14)
**COVID-19 (59)**	75.28 (20.45–69.88)	81.3% (49)	51.6% (31)
**Gastritis, unspecified (56)**	24.84 (8.03–29.92)	6.4% (26)	14.3% (8)
**Calculus of lower urinary tract, unspecified (55)**	50.29 (12.19–74.07)	50% (33)	34.5% (19)
**Obstructed labour due to unspecified causes (57)**	42. (18.79–32.30)	78.9 (45)	15.8% (9)
**Preterm labour or delivery, unspecified (51)**	39.63 (14.55–44.43)	74.5% (38)	25.5% (13)

#### Determinants of catastrophic health expenditure

The individual regression models revealed the following factors to be significantly associated with the likelihood for a patient to experience catastrophic health expenditure due to the costs of medication and consumables in our sample: gender, type of treatment received, facility type, and age.

In our sample, women were significantly more likely to experience CHE (estimate = 0.31565, z = 7.60, p < 0.001), as were patients who received inpatient non-surgical (estimate = 3.12384, z = 53.64, p < 0.001), and surgical care (estimate = 5.86042, z = 26.84, p < 0.001). Higher age (estimate = 0.0172, z = 15.38, p < 0.001) was associated with a higher likelihood of experiencing CHE, whereas treatment at a private facility was associated with a lower likelihood of experiencing CHE (estimate = -1.27921, z = -18.213, p < 0.001).

In the multiple regression analysis, all factors but male gender remained significant predictors of catastrophic health expenditure. The results of the multiple regression analysis are presented in Table 6 below.

**Table 6:** Results of a multiple logistic regression analysis examining the relationship between age, gender, treatment type (inpatient non-surgical care, surgical care, and outpatient care), and facility type (private, public and faith-based) and catastrophic health expenditure in a sample of 9,855 patients in the south of Madagascar. P-values below 0.05 were considered statistically significant.

Variable	Estimate	Standard Error	z-value	p-value
Age	0.021	0.001	12.859	< 0.001
Male sex	0.081	0.063	1.287	0.198
Inpatient non-surgical treatment	3.204	0.065	49.043	< 0.001
Surgical treatment	6.503	0.254	25.590	< 0.001
Private facility	-0.138	0.068	-2.017	0.043
Public facility	-1.604	0.127	-12.617	< 0.001

## Discussion

Our study revealed several important findings on patient costs and catastrophic health expenditure in our sample of 15 health facilities in Southern Madagascar.

Firstly, our research raises concerns about the financial burden associated with surgical care. We observed a high likelihood of CHE for patients undergoing surgical interventions in our sample, consistent with previous reports from the region [13]. For all cases of surgical interventions, more than 90% of patients suffered CHE at the 10% threshold. Given that this analysis only included costs for medication and consumables the actual rate of CHE in the sample is likely even higher. This underscores the urgent need for effective financial risk protection measures for patients needing surgical care.

Secondly, several patient groups requiring inpatient non-surgical care frequently experienced CHE in our sample: patients suffering from strokes, COVID-19, hypertension, and tuberculosis (TB). As routine care for non-communicable diseases was not included in the intervention’s eligibility criteria, patients were only eligible for inclusion if they suffered an exacerbation of their underlying diseases, such as a stroke or hypertensive crisis. It is possible that many patients whose diagnosis was described as “hypertension” in our sample were experiencing hypertensive crises, which was however inadequately described by the treating healthcare providers in the original reimbursement claims. However, given the rise in non-communicable diseases, especially stroke and hypertension across Sub-Saharan Africa, including Madagascar, and the relative lack of attention and resources these diseases have received in policy-making and financial risk protection in the country, these findings are worrying and indicate a patient group requiring further attention in financial risk protection schemes in the future. Especially in the absence of comprehensive access to long-term primary and secondary preventive care for patients with hypertension in Madagascar, which heightens the likelihood of experiencing acute exacerbations and complications of the disease.

Thirdly, our analysis revealed differences in patient costs across types of healthcare facilities, with private facilities having the lowest costs and faith-based facilities the highest. This variation was likely due to the different scope of services offered. Private facilities mostly provided outpatient services which are generally less costly than inpatient or surgical care. Faith-based facilities served as major surgical referral centers, where surgical procedures are more expensive. Public facilities offered both inpatient and outpatient care.

Further, in our sample, 360% and 33% of people with TB who accessed care experienced CHE at the 10% and 25% threshold, respectively. This is particularly worrying as people with TB are often among the poorest strata of society [25] and face social exclusion due to the stigma associated with TB [26], rendering them unable to work during their illness, and further worsening their socioeconomic situation [26]. Our findings suggest that policy and practice currently fall short of effectively safeguarding this vulnerable population from CHE.

Lastly, our study showed that costs for maternity care were significantly higher on average than for any other type of care. This is likely because many cases accessing treatment for maternal care required surgical treatment, most commonly Caesarean sections and because we included many secondary facilities in our sample that are more likely to treat complicated cases of maternity care and those requiring surgical intervention. However, the fact that maternity care, albeit predominantly at a secondary care level, is significantly more expensive than other types of care provided at the same facilities is worrying, given the pre-existing economic and social disadvantages faced by women in Southern Madagascar, as they are less likely to earn a living wage and often have limited control over their finances [11]. In this context women are particularly ill-placed to pay for elevated costs of care and may thus be at a particular risk of experiencing CHE. Maternity care at secondary facilities and for complicated cases should thus be a particular focus of financial risk protection efforts of both governmental and non-governmental actors in the region in the future to prevent CHE among women, improve their socioeconomic standing and contribute to gender equity in the long term.

While our study provides valuable insights, it is not without limitations. Firstly, our analysis focused solely on the costs of medication and consumables. Other costs such as costs for hospitalisation and diagnostic services were not included in our study, which might have underestimated the rate of catastrophic health expenditure, especially among patients who received inpatient services.

Secondly, our expenditure data is based on individual-level expenditure data and average household size from the 2010 census, as this is the last data source citing comprehensive expenditure data for all regions [21]. Although poverty rates in the region remained relatively stable [27], there may have been some changes in average household expenditure over time that we could not capture in our study. Additionally, the absence of individual-level data for household income and expenditure necessitated the use of modelled estimates for CHE. It is possible that households and individuals included in this study sample differed from the regional average in household size or expenditure, which we would fail to capture. However, as we used a representative, nationwide survey to estimate household size and expenditure [21], we are confident these modelled estimates provide a robust representation of the actual situation.

Further, we are measuring the cost of care and CHE from data of an intervention that specifically targets costs of care and CHE. This might have affected the costs of medical care, for example through an influence on prescribing patterns, and thus the prevalence of CHE in both directions.

Lastly, our study included data from a convenience sample of facilities in Southern Madagascar, which are not representative of the general health system in the country. However, our sample included a wide selection of private sector and faith-based facilities, which account for over 35% of health services delivered in Madagascar but for which data on costing and financial risk protections are particularly scarce [7].

To address the limitations of our study, future research in the area should incorporate individual-level income data and include additional costs outside of medications and consumables. These refinements can provide a more comprehensive and accurate assessment of the prevalence and factors contributing to CHE in southern Madagascar.

## Conclusion

Our study revealed that patient costs in our study sample were likely to be catastrophic, especially for patients requiring in-patient and surgical care. Our findings underscore the importance of financial risk protection for patients accessing care for maternity care, paediatric care, accidents and injuries and acutely life-threatening conditions in Madagascar Further, our study shows that the high costs of maternity care for complicated cases merit particular attention and require the consideration of gender-specific differences in financial risk protection interventions.
